# Minimal risk of *Rabies lyssavirus* spillover from bats in Israel: findings from long-term surveillance

**DOI:** 10.3389/fmicb.2025.1707522

**Published:** 2025-12-08

**Authors:** Maya Weinberg, Roni King, Roi Lapid, Boris Yakobson, Marina Eyngor, Dongsheng Luo, Efstathios S. Giotis

**Affiliations:** 1 School of Zoology, Faculty of Life Sciences, Tel Aviv University, Tel Aviv, Israel; 2 School of Life Sciences, University of Essex, Colchester, United Kingdom; 3 Israel Nature and Parks Authority, Jerusalem, Israel; 4 Department of Veterinary Medicine, University of Cambridge, Cambridge, United Kingdom; 5 Koret School of Veterinary Medicine, The Hebrew University of Jerusalem, Rehovot, Israel; 6 The Rabies Laboratory, Kimron Veterinary Institute, Bet Dagan, Israel; 7 Department of Infectious Diseases, Imperial College London, London, United Kingdom

**Keywords:** *Lyssavirus*, rabies, bats, cross-species transmission, risk

## Abstract

Rabies, caused by viruses of the genus *Lyssavirus* (family Rhabdoviridae), remains a persistent public health and veterinary challenge in Israel, a small but strategically positioned country at the crossroads of Africa, Asia, and Europe. Over the past 18 years (2006–2024), more than 670 confirmed rabies cases have been reported in humans, wildlife, farm animals, and stray dogs, with the latest human fatality recorded in November 2024, following a bite from an unvaccinated dog. While domestic dogs (*Canis familiaris*) and golden jackals (*Canis aureus*) account for the majority of cases, little is known about the role of Israel’s 32 bat species in the circulation of *Lyssavirus rabies*. We analyzed national rabies testing records spanning nearly three decades (1995–2022), encompassing over 42,000 animals. Among the 294 bats tested, representing both insectivorous and frugivorous species, none were positive for rabies virus (*R. lyssavirus;* RABV). These findings contrast sharply with the high prevalence detected in carnivores and livestock and align with the absence of documented bat-to-human transmission events in Israel. These results suggest that bats in Israel remain largely isolated from the *Lyssavirus* transmission cycles affecting other domestic and wild mammals and may pose minimal risk for rabies or other *Lyssavirus* spillover in the region. They also underscore the significance of sustained long-term surveillance to detect emerging *Lyssavirus* threats.

## Introduction

Rabies, caused by viruses of the genus *Lyssavirus* (family *Rhabdoviridae*), remains a major zoonotic threat worldwide and is almost invariably fatal once clinical symptoms appear (Johnson et al., 2010; Hampson et al., 2015). The rabies virus (*Rabies lyssavirus*, genotype 1; RABV) is primarily transmitted through the bite of infected animals, most commonly domestic dogs, and continues to cause tens of thousands of human deaths annually (Hampson et al., 2015). Bats are frequently perceived as high-risk reservoirs for rabies because they serve as natural hosts for multiple *Lyssavirus* species globally; however, their true epidemiological role varies markedly between regions. These lyssaviruses show distinct geographic distributions and are often associated with specific bat hosts (Johnson et al., 2010). While bat-associated lyssaviruses are capable of infecting humans and other mammals, cross-species transmission events remain rare (Wallace et al., 2014).

Notably, of the 18 recognized lyssaviruses, RABV is the only species found in the Americas, and to date, no bats outside this region have been reported to be infected with RABV (Markotter and Coertse, 2018). Cases of RABV related to bats are most frequently reported in Latin America, with fewer occurrences in the United States, where the virus is present across multiple bat species (Marston et al., 2018). From a host-pathogen perspective, the New World bat RABV has been linked to over 40 different species of insectivorous, sanguivorous, and frugivorous bats. Consequently, New World bat populations serve as a constant source of RABV infections, for which efficient mitigation measures are currently unavailable (Marston et al., 2018).

In contrast, RABV is absent in Old World bats but circulates among carnivores, while other bat-associated lyssaviruses are restricted to the Old World (Troupin et al., 2016; Hayman et al., 2016). Instead, insectivorous and frugivorous bat species in the Old World seem to serve as reservoirs for to non-RABV *lyssaviruses* (Banyard et al., 2013). They are often suspected of facilitating cross-species transmission of viruses from other families. However, actual transmission in many cases was not sufficiently supported (Weinberg and Yovel, 2022). Six bat-related lyssaviruses have been identified in the European region, including *Kotalahti bat lyssavirus* (KBLV) in Finland, *Lleida bat lyssavirus* (LBLV) in Spain and France, and *Bokeloh bat lyssavirus* (BBLV) in several central and western European countries (Smreczak et al., 2018; Picard-Meyer et al., 2019; Ceballos et al., 2013; Calvelage et al., 2021). *European bat lyssavirus 2* (EBLV-2) has been detected only in Western and Northern Europe, while *West Caucasian bat virus* (WCBV) has been found in the western Caucasus region of Russia (McElhinney et al., 2018; Kuzmin et al., 2008). Among these, *European bat lyssavirus 1* (EBLV-1) has the widest distribution, having been detected in almost all European countries (Marston et al., 2018; McElhinney et al., 2013). Indeed, the majority of bat-related lyssavirus cases are linked to *European Bat Lyssavirus Type 1* (EBLV-1; Schatz et al., 2013). However, the detection of highly divergent lyssaviruses, such as *Lleida bat lyssavirus* (LLEBV; Banyard et al., 2018) and *West Caucasian bat lyssavirus* (WCBV) in Europe, and the potential *Matlo bat lyssavirus* (MBLV; Coertse et al., 2020) in the Republic of South Africa, poses an increasing risk of cross-species transmission to both human and animal populations. This risk is particularly concerning, given the lack of licensed biologics with efficacy against these *Lyssavirus* species (Servat et al., 2019). In terms of bat–host associations, all of these lyssaviruses were identified in insectivorous bats, particularly those belonging to the *Myotis* and *Miniopterus* genera (McElhinney et al., 2013). The latter comprises more than 30 named species and is widespread in the Old World (Miller-Butterworth et al., 2007). These bats exhibit a broad distribution range, with 46 distinct lyssaviruses documented, including LLEBV and WCBV. Evidence also suggests both geographical distribution and compartmentalized co-evolution between bat species and specific *Lyssavirus* species (Fooks et al., 2021). The significance of bat rabies for public health in Europe is highlighted by the fact that both EBLV-1 and EBLV-2 have caused human fatalities, although the number of such cases is limited (Schatz et al., 2013). However, no bat-related lyssaviruses have been reported in geographically nearby central eastern countries such as Israel (Weinberg and Yovel, 2022; Banyard et al., 2020).

Israel is a small but strategically situated country at the crossroads of Africa, Asia, and Europe, with rabies remaining endemic in terrestrial mammals. Over the past two decades, more than 670 confirmed RABV cases have been reported in dogs, jackals, foxes, and other mammals (Fooks et al., 2021). However, the contribution of Israel’s 32 bat species to *Lyssavirus* circulation remains poorly studied. The Egyptian fruit bat (*Rousettus aegyptiacus*), the country’s only frugivorous bat and, to date, the most abundant species, is found close to the habitat of humans and domestic animals. It frequently forages in urban environments, increasing opportunities for interaction with pets, stray dogs and cats, and synanthropic wildlife such as rats and crows, some of which are known predators of bats and confirmed RABV hosts (Banyard et al., 2018; Coertse et al., 2020; Servat et al., 2019). Despite this proximity, no cross-species transmission events from bats to humans or terrestrial mammals have ever been documented in Israel, raising questions about their role in the country’s rabies epidemiology.

A common argument is that the absence of reported *Lyssavirus* infections in bats reflects a lack of surveillance rather than true absence. To address this, the study analyzed nearly three decades (1995–2022) of national rabies testing records encompassing more than 42,000 animals, including 294 insectivorous and frugivorous bats. It aimed to evaluate the prevalence of RABV in bats in Israel relative to other mammalian species and assess their potential role in *Lyssavirus* spillover. This analysis provides the most extensive evidence to date regarding bat-associated to non-RABV *lyssaviruses* in Israel and contributes to the broader understanding of rabies dynamics in Old World bat populations.

## Materials and methods

### Data source

We reviewed records from the Israeli Veterinary Institute’s rabies testing database, which documents over 42,000 tests conducted from 2006 onwards. Additional data on bats and other mammals tested for rabies between 1995 and 2022 were obtained from the Israel Nature and Parks Authority (INPA) and the National Veterinary Archive (Supplementray Data).

### Sampling

Mammals are routinely tested for rabies in Israel, either as part of post-mortem diagnostic investigations or wildlife surveillance programs. Wild animals, including bats, are primarily sampled through the Israel Wildlife Disease Surveillance Program (IWDS). These samples include apparently healthy bats culled during routine monitoring and bats found to be sick, injured, or dead. Farm and companion animals are generally tested when showing neurological signs consistent with rabies.

A total of 294 bats were tested between 1998 and 2022. Of these, 240 out of 294 (82%) were insectivorous bats and could not be identified to the species level, while the remaining 54 (18%) were Egyptian fruit bats (*Rousettus aegyptiacus*). A total of 74 bats (25%) were examined as part of IWDS wildlife surveillance, and the remaining bats were tested because they were sick, injured, or found dead.

### Rabies testing

All samples were tested for rabies virus (RABV) by the Department of Rabies at the Israeli Veterinary Institute using the Touch Impression Fluorescent Antibody Test (TAF)—the historical gold-standard diagnostic method recommended by the WHO and World Organization for Animal Health (OIE; Mayes and Rupprecht, 2015; World Health Organization, 2018). Brain tissue from the samples was examined for the presence of rabies antigen using fluorescently labeled antirabies antibodies, and positive samples were confirmed through fluorescence microscopy. Until 2014, bat samples were also tested using virus isolation in cell culture. In cases where the brain tissue was unsuitable for antigen detection, real-time PCR was employed as an alternative diagnostic method. For suspected human rabies cases, saliva, cerebrospinal fluid (CSF), and skin biopsy were analyzed using real-time PCR as the standard diagnostic assay. When a positive result from any mammal was obtained, conventional PCR was subsequently performed to amplify a larger fragment of the nucleoprotein (N) gene, enabling phylogenetic analysis to identify the viral genetic variant and determine its geographic or host origin.

### Data analysis and visualization

Data on rabies test outcomes were analyzed in R software (version 4.3.0; R Foundation for Statistical Computing, Vienna, Austria) using base functions along with the reshape2, ggplot2, and patchwork packages. Descriptive statistics were calculated for each animal species, and the prevalence was calculated as the proportion of positive cases among all samples tested. Stacked bar plots were used to visualize temporal trends and comparisons between tested and positive cases across species and years. A segmented y-axis was used for high-volume species to improve interpretability. All figures were generated in R and formatted for publication using Adobe Illustrator.

To illustrate the geographic distribution of bats examined in this study, we mapped 220 cases with available location data out of 294 total samples. The map was generated using the open-access simplemaps platform.

## Results

**Figure 1:**
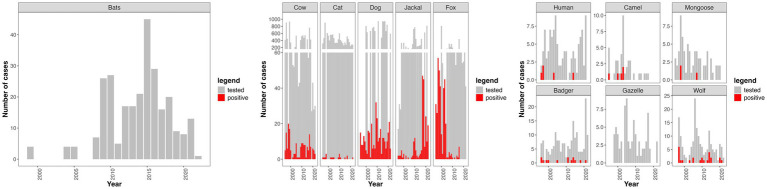
Three-panel figure showing trends in rabies testing and confirmed cases across multiple species from 1995 to 2022. The x-axis represents years, and the y-axis represents the number of cases, with species-specific y-axis scales to enhance clarity. Gray bars indicate the number of samples tested, and red bars indicate the subset confirmed positive for rabies. Panel 1 shows data for cows, cats, dogs, jackals, and foxes. Panel 2 shows data for badgers, gazelles, wolves, humans, camels, and mongooses. Panel 3 shows data for bats.

During the study period, over 670 confirmed cases of RABV in wildlife, farm animals, and stray dogs were recorded in Israel, with a total annual average of 37 cases across many species. The majority of these cases were identified in dogs (*Canis familiaris*) and golden jackals (*Canis aureus*), but RABV-positive cases were also reported in various other mammals, including cattle (*Bos taurus*), sheep (*Ovis aries*), horses (*Equus caballus*), wolves (*Canis lupus*), foxes (*Vulpes vulpes*), badgers (*Meles meles*), beech martens (*Martes foina*), cats (*Felis catus*), and rats (*Rattus rattus*) (Figure 1).

**Figure 2:**
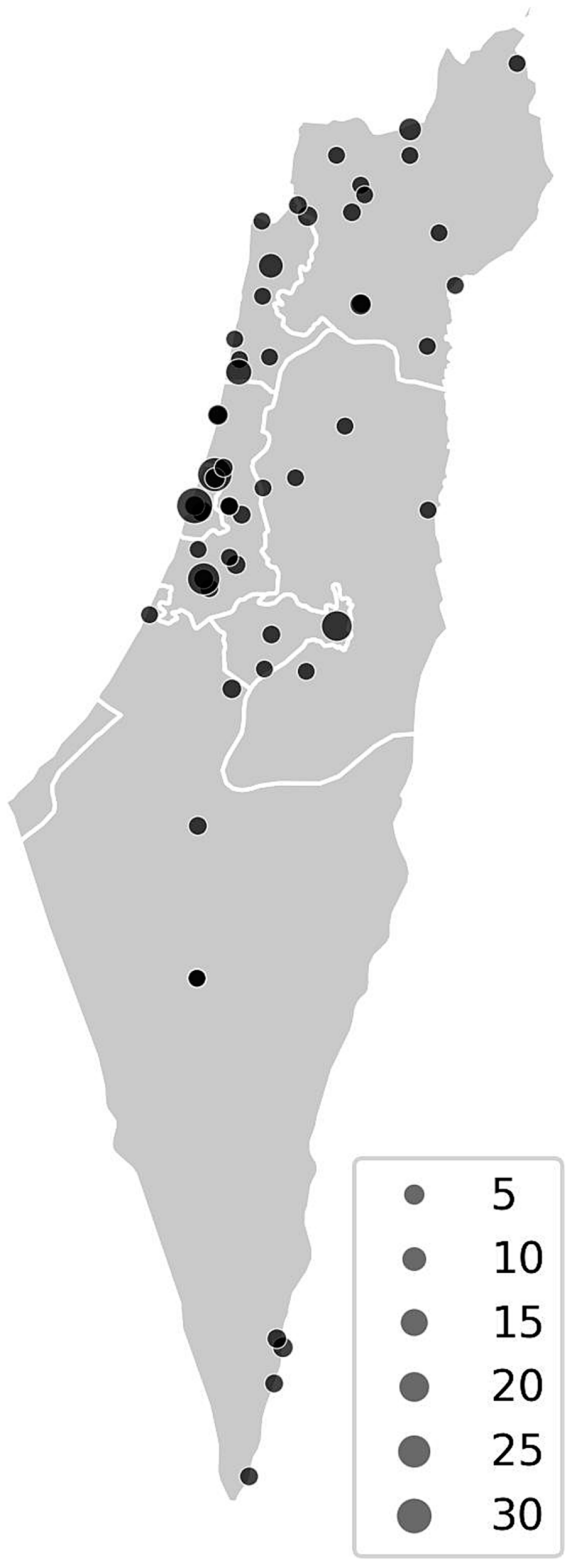
Schematic geographic distribution of 220 bat samples examined for rabies out of 294 total samples included in this study. Each point represents a sampling location, and point size corresponds to the number of samples recorded at that site. No positive cases were detected.

Among the sampled animals, RABV prevalence was the highest in foxes (350 positive cases out of 2,707 tested; 13%), wolves (25 out of 151; 16%), and martens (5 out of 39; 13%). In terms of absolute numbers, irrespective of sampling proportions, the highest number of RABV cases were recorded in foxes (350), dogs (323), jackals (213), and cows (176). Furthermore, the bat dataset spans 24 years (1998–2022) and includes 294 bats tested for RABV across 49 locations under 10 different veterinary departments, covering both northern and southern regions of Israel (Figure 2, map). According to this dataset, no bats tested positive (Figure 1).

## Discussion

Our findings, based on nearly three decades of rabies virus (RABV) surveillance in Israel, provide no evidence of *Lyssavirus* infection in bats. Despite extensive testing of 294 bats between 1998 and 2022, including species frequently found in proximity to humans, no RABV cases were detected. This contrasts with the consistent detection of RABV in domestic dogs, jackals, foxes, and other terrestrial mammals (David et al., 1999) and supports the view that bats in Israel pose minimal risk for rabies spillover.

This result reflects the broader epidemiological distinction between bats of the Old and the New World. In the Americas, RABV is enzootic in multiple bat taxa and is a major source of human and livestock infections (Letko et al., 2020; Rupprecht et al., 2017). More than 40 bat species serve as reservoirs, facilitating frequent spillover and, in some rare cases, host-switching events that enable onward transmission (Shipley et al., 2019). In contrast, Old World bats have never been shown to maintain RABV, which instead circulates predominantly among carnivores (Troupin et al., 2016; Hayman et al., 2016; Banyard et al., 2020). The Old World bat species harbor a variety of non-RABV lyssaviruses, such as EBLV-1, EBLV-2, LLEBV, and WCBV, most of which exhibit apparent host restriction and low spillover potential (Banyard et al., 2018; Coertse et al., 2020; Servat et al., 2019; Schatz et al., 2013; Dundarova et al., 2023; McElhinney et al., 2018). This pattern may be linked to long-term virus-host co-evolution, narrower host range, and species-specific ecology (Shipley et al., 2019; Leopardi et al., 2025).

Interest in bats as reservoirs of zoonotic viruses has increased globally due to the ongoing detection of novel lyssaviruses and other pathogens, such as coronaviruses and filoviruses (World Health Organization, 2018). Although *Lyssavirus* transmission from bats to terrestrial species is rare, it remains significant because of the invariably fatal nature of rabies (Johnson et al., 2010; Hampson et al., 2015; World Health Organization, 2018). The recent emergence of highly divergent viruses, such as LLEBV and Lloviu virus in Europe, highlights the need for ongoing surveillance of emerging/novel bat-borne lyssaviruses, particularly given the limited cross-protection that current rabies vaccines provide against phylogroup II and III lyssaviruses (Servat et al., 2019; Echevarría et al., 2019).

While our sample size provided 95% power to detect infections at a prevalence of ≥1%, the absence of positive detections does not fully rule out the possibility of low-level circulation. Limitations of the study include uneven sampling across years, locations, and species. Additional constraints include the inherently limited number of wildlife submissions, particularly for bats, and the lack of species-level identification, reflecting passive rather than systematic surveillance. Data prior to 1995 were not digitized and therefore could not be retrieved for analysis, thereby restricting the assessment of longer-term trends. During most of the study period, the golden standard diagnostic approach did not include molecular typing or genomic analyses, which explains the absence of such data in this dataset, thereby limiting the ability to distinguish between viral lineages and assess the transmission dynamics in greater detail (Leopardi et al., 2025; Hanlon and Nadin-Davis, 2013; Fischer et al., 2014). In subsequent analyses, all rabies-positive brain samples collected between 1995 and 2025 were subjected to phylogenetic characterization, revealing exclusive affiliation with *Rabies lyssavirus*, genotype 1; (RABV), with no evidence of bat-associated lyssavirus genotypes (David and Yakobson, 2011; Ministry of Agriculture and Food (Israel), 2005).

Expanded active surveillance using molecular approaches such as RT-qPCR for viral RNA and broad serological studies would enhance our ability to detect rare infections and better characterize potential spillover risks (Leopardi et al., 2025; Fischer et al., 2014; Echevarría et al., 2001).

Overall, our data support the conclusion that bats in Israel do not currently play a role in RABV maintenance and pose a minimal spillover risk. However, as dog-mediated rabies declines globally, the relative significance of bat *Lyssaviruses* may increase (Echevarría et al., 2001).

Rabies caused by classical RABV remains a major public health and veterinary concern in Israel, with cases regularly reported in wildlife, livestock, and companion animals. In Israel, the majority of recent rabies cases are detected in wild canids, particularly jackals. Slightly fewer cases are diagnosed in dogs, which still play a considerable role in transmission, especially in rural and border areas. Cases in foxes and wolves have become very rare in recent years. Occasional spillover to domestic herbivores, including cattle, sheep, and horses, is also observed. Given its clear epidemiological dominance and impact in the region, this study focused on RABV as the primary agent of concern. Nevertheless, future surveillance and continuous monitoring should also include broader *Lyssavirus* screening to ensure early detection of potential emerging threats and to assess vaccine efficacy against divergent strains.

Despite the limitations outlined above, bats have been included in national rabies surveillance and pathological examinations over the years, as documented in our dataset. This study compiles these surveillance records for the first time and shows that, although bats were regularly tested, no RABV-positive cases have been recorded to date.

## Data Availability

The original contributions presented in the study are included in the article/Supplementary material; further inquiries can be directed to the corresponding author.

## Supplementary material

DATA SHEET 1 All sampled animals tested for rabies virus (RABV).

DATA SHEET 2 All sampled animals that tested positive for rabies virus (RABV).
